# Effects of Ferrocenyl 4-(Imino)-1,4-Dihydro-quinolines on *Xenopus laevis* Prophase I - Arrested Oocytes: Survival and Hormonal-Induced M-Phase Entry

**DOI:** 10.3390/ijms21093049

**Published:** 2020-04-26

**Authors:** Guillaume Marchand, Nathalie Wambang, Sylvain Pellegrini, Caroline Molinaro, Alain Martoriati, Till Bousquet, Angel Markey, Arlette Lescuyer-Rousseau, Jean-François Bodart, Katia Cailliau, Lydie Pelinski, Matthieu Marin

**Affiliations:** 1 CNRS, UMR 8576-UGSF-Unité de Glycobiologie Structurale et Fonctionnelle, Univ. Lille, F-59000 Lille, France; 2 CNRS, Centrale Lille, Univ. Artois, UMR 8181-UCCS-Unité de Catalyse et Chimie du Solide, Univ. Lille, F-59000 Lille, France

**Keywords:** ferrocenyldihydroquinoline, *Xenopus laevis*, oocyte, metaphase II spindle, cell death

## Abstract

*Xenopus* oocytes were used as cellular and molecular sentinels to assess the effects of a new class of organometallic compounds called ferrocenyl dihydroquinolines that have been developed as potential anti-cancer agents. One ferrocenyl dihydroquinoline compound exerted deleterious effects on oocyte survival after 48 h of incubation at 100 μM. Two ferrocenyl dihydroquinoline compounds had an inhibitory effect on the resumption of progesterone induced oocyte meiosis, compared to controls without ferrocenyl groups. In these inhibited oocytes, no MPF (Cdk1/cyclin B) activity was detected by western blot analysis as shown by the lack of phosphorylation of histone H3. The dephosphorylation of the inhibitory Y15 residue of Cdk1 occurred but cyclin B was degraded. Moreover, two apoptotic death markers, the active caspase 3 and the phosphorylated histone H2, were detected. Only 7-chloro-1-ferrocenylmethyl-4-(phenylylimino)-1,4-dihydroquinoline (**8**) did not show any toxicity and allowed the assembly of a histologically normal metaphase II meiotic spindle while inhibiting the proliferation of cancer cell lines with a low IC_50_, suggesting that this compound appears suitable as an antimitotic agent.

## 1. Introduction

Among ecosystems, the aquatic compartment is particularly impacted by contaminants as it constitutes the final receptacle of dispersion, runoff, and spills. For decades, amphibians have been regarded as key species for gathering new knowledge and discovering original concepts, which have then been extended to other animal models. Their well-known physiology, development and reproduction can be easily exploited in aquatic ecotoxicology and their highly-conserved molecular mechanisms compared to humans represent an important advantage [1,2,3]. The South African clawed frog *(Xenopus laevis*) is one of the most valuable sentinel species for accessing environmental health effects [4] caused by any direct exposure to environmental micropollutants in the aquatic compartment on which they depend [5]. If the effects of micropollutants have often been tested on early developmental stages (Frog Embryo Teratogenesis Assay - *Xenopus*, FETAX) [6], only few studies have determined their impacts on reproduction, oogenesis and meiosis completion.

In anticipation of zygote formation, and the restoration of diploidy, *Xenopus* ovaries produce oocytes arrested in prophase I in a state analogous to the G2-phase of the cell cycle. *Xenopus* oocytes can therefore be used to study cell cycle signaling [7,8,9] at the single cell level [10,11] and to carry out toxicity analyzes [3,12,13]. Immature oocytes that are blocked in prophase I resume their meiosis upon hormonal stimulation, mimicking the steroid stimulation provided by the in vivo follicular cells. Meiotic resumption, considered as an M-phase entry, is characterized, at the morphological level, by the occurrence of a white spot at the oocyte animal pole. This white spot is a hallmark for oocyte progression from meiosis I to metaphase II, and attests the migration and the dissolution of the germinal vesicle (also called germinal vesicle breakdown, GVBD). The migration of the nuclear material towards the animal pole is followed by chromosome condensation, the formation of a meiotic spindle anchored to the plasma membrane and a polar body extrusion. Metaphase II oocytes are blocked in anticipation of fertilization [14]. At the molecular level, meiotic resumption is triggered by the activation of the universal heterocomplex Cdk1/cyclin B, also called MPF (M-Phase Promoting Factor), and by Cdc25 [14]. In parallel to MPF, MAPK/Erk activation [15] is required for proper maturation including meiotic spindle formation [16,17] and the absence of DNA synthesis between the two meiotic divisions [18].

**Figure 1:**
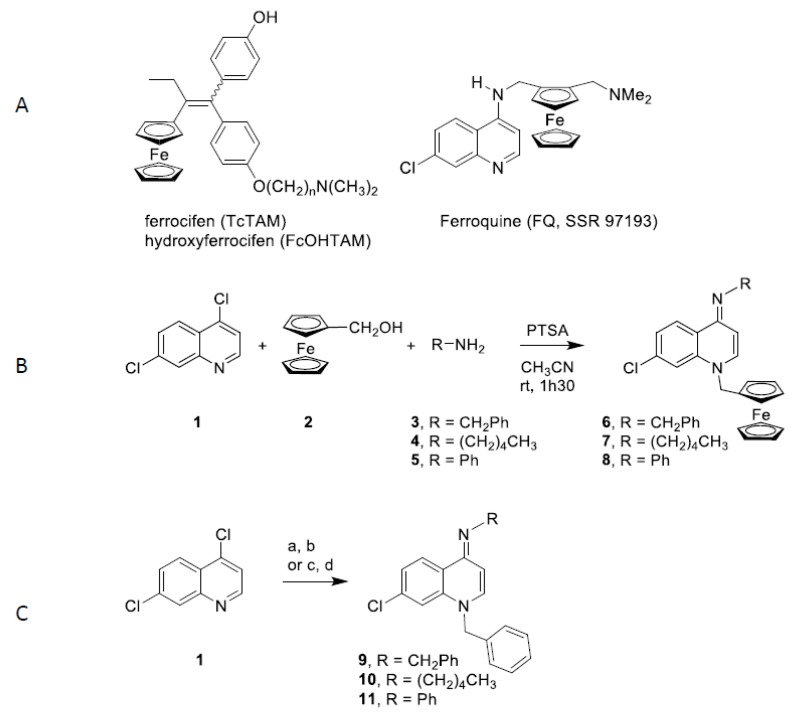
Structure and synthesis of 4-(imino)-1,4-dihydroquinoline derivatives. **A**. Structures of ferrocifen, hydroxyferrocifen and ferroquine; **B**. Synthesis of ferrocenyl 4-(imino)-1,4-dihydroquinolines **6**-**8**. **C**. Synthesis of benzyl 4-(imino)-1,4-dihydroquinolines **9** and **10**: (a) benzyl chloride, NaI, acetone, reflux, 12 h; (b) RNH_2_, EtOH, relux, 1 h. Synthesis of benzyl 4-(imino)-1,4-dihydroquinolines **11**: (c) aniline, microwaves, 100 °C, 10 min; (d) benzyl chloride, microwaves, 80 °C, 40 min.

The design and the synthesis of new efficient molecular agents against cell cycle deregulation is a major objective of current biology today due to the perpetual adaptation and resistance of cellular processes. Organometallic compounds have become a relevant strategy of research in the field of chemical biology. A particularly interesting core, (imino)-1,4-dihydroquinoline, appears increasingly in compounds of therapeutic importance such as hypotensive agents [19] or inhibitors of ion channels [20]. In addition, quinoline-4(1*H*)-imines is a potent antiplasmodial targeting the liver stage of malaria [21]. Their physicochemical and electrochemical properties offer new possibilities in therapeutic applications [22]. In particular, a number of metal base drugs display interesting activities as anticancer, antimicrobial and antimalarial agents [23,24,25]. Among them, ferrocifen (TcTAM) and hydroxyferrocifen (FcOHTAM) constitute the first iron-based organometallic derivatives that demonstrate a high in vitro antiproliferative activity on both hormono-dependent (MCF-7) and hormono-independent (MDA-MB-231) breast cancer cell lines [26,27,28]. This strategy was also used for ferroquine (FQ, SSR 97193), a ferrocenyl analogue of the antimalarial chloroquine that also shows anticancer activity is in clinical phase 2 trials [29,30] (Figure 1A). While certain mechanisms of action and targets have been described, including DNA breaks and enzymes involved in cell cycle regulation [23,24,29], they are still under investigation. As part of an ongoing effort to develop new organometallic drugs, [31,32,33] we have previously reported an easy and rapid method for synthesis of ferrocenyl(imino)-1,4-dihydroquinolines through a novel multicomponent reaction [34].

Pharmaceuticals can enter the aquatic environment by excretion of treated patients, through hospital wastewater and pharmaceutical industries. Concentrations of pharmaceuticals detected in water are in the range from ng/L to μg/L. Their frequent use produces a continuous supply in the environment, accumulations in living organisms and serious impacts [35,36]. As a consequence, the toxicity and the possible contribution of new drugs to an environmental contamination need to be tested. In the present study, we used *Xenopus laevis* oocytes to evaluate the effect of dihydroquinolines on cell survival and meiosis progression in metaphase II. Prophase I arrested oocytes and metaphase II entry were evaluated using a phenotypic approach and western blot analysis of cell cycle and cell death markers and the metaphase II meiotic spindle was visualized on histological sections.

## 2. Results

### 2.1. Synthesis of (imino)-1,4-Dihydroquinoline Derivatives

The ferrocenyl imino-1,4-dihydroquinolines **6**-**8** were synthesized through a multicomponent reaction according to Figure 1B. Condensation of 4,7-dichoroquinoline (**1**), ferrocenyl alcohol **2** and amines **3**-**5** in presence of *p*-toluenesulfonic acid (PTSA) provided the ferrocenyl imino-1,4-dihydroquinolines **6**-**8** in 67–82% yields. Following a described procedure [37], imino-1,4-dihydroquinolines **9** and **10** were obtained in 20% and 27% global yields in two steps: condensation of benzyl chloride on 4,7-dichloroquinoline (**1)** and treatment with amines **3** and **5** (Figure 1C). Imino-1,4-dihydroquinoline **11** was synthesized using a microwave procedure whereby a nucleophilic substitution of chloride by aniline was followed by the action of benzyl chloride to afford compound **11** in 28% global yield (Figure 1C).

### 2.2. Effect of Ferrocenyl 4-(imino)-1,4-Dihydroquinolines on Oocyte Survival

**Figure 2:**
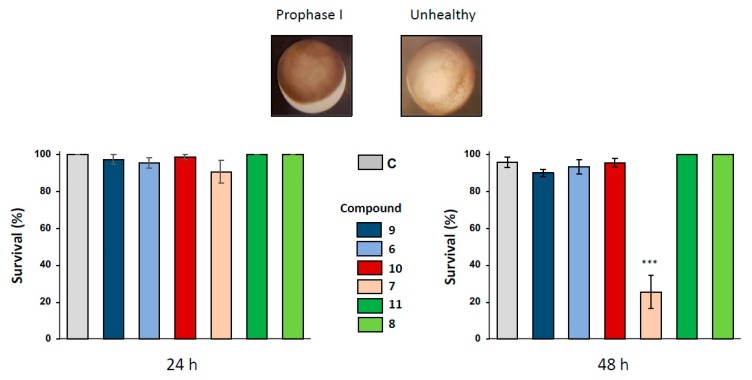
Effect of ferrocenyl 4-(amino)-1,4-dihydroquinolines exposures on oocytes survival. Oocytes were exposed up to 48 h to 100 µM of each compound (**9** in dark blue, **6** in light blue, **10** in red, **7** in pink, **11** in dark green and **8** in light green). After 24 h and 48 h of exposure, oocytes survival was assessed by phenotypical approach. As depicted, healthy oocytes (named as prophase I) presented a brown homogenous pigmentation compared to unhealthy one. All the results were compiled and compared to control (ND96, in grey) conditions. Statistical significance was assessed by a two-way ANOVA followed by Dunnett’s test, *** *P* < 0.001. *N* refers to the number of females and *n* to the number of oocytes (*N* = 3 and *n* = 60).

The effects of 1,4-dihydroquinoline derivatives were first assayed on *Xenopus* oocytes arrested in prophase I after 24 and 48 h of exposure to the ferrocenyl 4-(imino)-1,4-dihydroquinolines **6-8** and their respective benzyl 4-(imino)-1,4-dihydroquinolines control structures **9-11** at concentrations of 1, 10, 50 and 100 µM. After 24 h of incubation with all the (imino)-1,4-dihydroquinolines, no significant phenotypical modification was observed in prophase I oocytes as demonstrated by the sample treated with the concentration of 100 µM (Figure 2). Healthy oocyte phenotypes are characterized by the presence of two hemispheres, a homogenous dark-brown pigmented animal hemisphere and a clearer vegetative hemisphere (Figure 2). Healthy oocytes were also controlled by recording the resting potential of treated and non-treated cells. Only compound **7** produced a depolarization of the membrane potential in 100 µM-treated oocytes (Supplementary Figure S1), near the potential measured in apoptotic cells.

Oocyte hemi-sections were made to ascertain the presence of an asymmetrically positioned germinal vesicle in the animal hemisphere. When a longer exposure period of 48 h was used with 1,4-dihydroquinolines at the concentration of 100 µM, only compound **7** provoked a significant altered pigmentation in the oocyte animal pole and induced oocyte death with a bleaching phenotype. No modifications of oocyte integrity were observed for the other 1,4-dihydroquinolines as compared to the untreated control (Figure 2).

**Figure 3:**
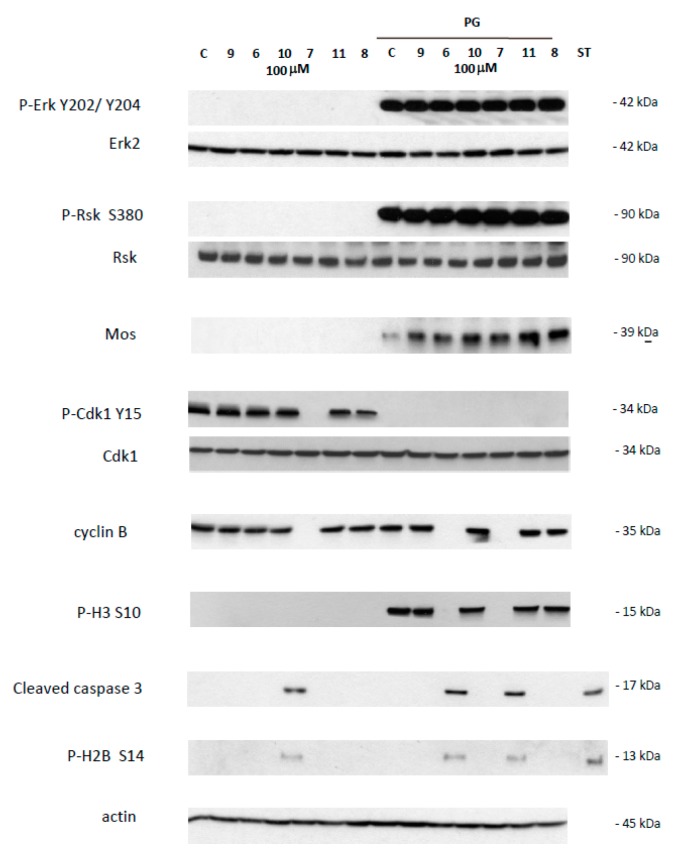
Western blot analysis of oocytes treated by 4-(imino)-1,4-dihydroquinolines. Oocytes untreated (C) or treated with progesterone (PG) were submitted to 4-(imino)-1,4-dihydroquinolines **6, 7, 8, 9, 10, 11** for 24 h. After electrophoresis, antibodies were used to reveal total and phosphorylated forms of Erk2 and P-Erk2 on Y202/Y204, Rsk and P-Rsk on S380, Cdk1 and P-Cdk1 on Y15, and phosphorylated form of P-H3 on S10, P-H2B on S14, or cleaved caspase 3, cyclin B2, Mos and actin. The membranes were revealed using an Advanced ECL chemiluminescence technology. Staurosporine 20 mM was used as apoptosis control. Erk2, Rsk, Cdk1 and actin serve as internal loading controls.

This effect was further confirmed by western blot analysis of samples 24 h after incubation with 1,4-dihydroquinolines (100 µM). In prophase I arrested oocytes, Erk2 and Rsk were unphosphorylated in all treated conditions as for the untreated control (Figure 3). Mos was not present and Histone H3 was not phosphorylated as expected. The inactive Y 15 phosphorylated form of Cdk1 and cyclin B were present, as seen in untreated control, except for the oocytes treated by compound 7. Two death markers for apoptosis, the cleaved caspase 3 and the phosphorylated histone H2, were detected only in oocytes exposed to compound **7**.

### 2.3. Effects of 1,4-Dihydroquinolines on Mitotic Cell Division

Two cancer cell lines, the MDA-MB-231 triple negative breast cancer cell line and the uterine Hela cells, were used to establish the toxicity of each 1,4-dihydroquinoline. The IC_50_ values necessary to induce somatic cell death ranged between 3.39 and 42.09 µM. The ferrocenyl 4-(imino)-1,4-dihydroquinolines **6, 7** and **8** had a lower IC_50_ compared to their respective benzyl 4-(imino)-1,4-dihydroquinolines **9, 10** and **11** devoid of ferrocene. Respectively, for the MDA-MB-231 and for the Hela cells, the fold decrease in the IC_50_ produced by the addition of a ferrocene group was 3.22 and 2.72 for compound **6**, 3.25 and 2.90 for compound **7**, and 1.88 and 6.77 for compound **8** (Table 1).

### 2.4. Effects of 1,4-Dihydroquinolines on Meiosis Resumption

**Figure 4:**
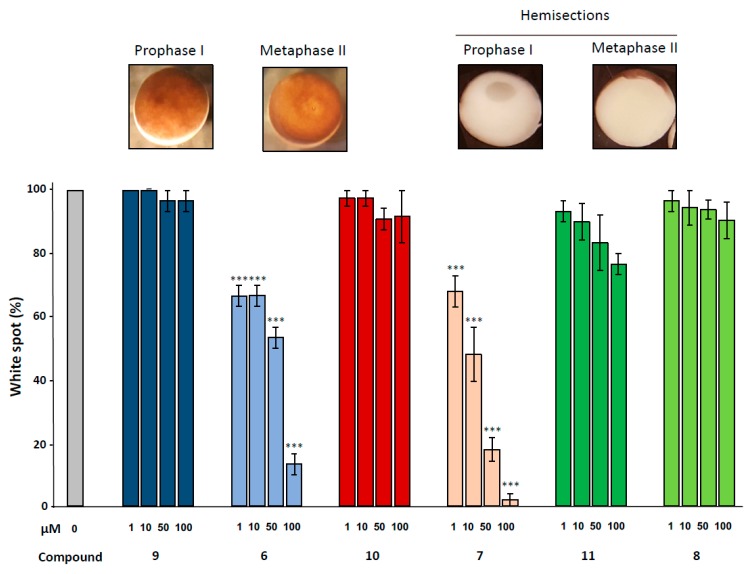
Effect of ferrocenyl 4-(alkylamino)-1,4-dihydroquinolines exposures on oocytes maturation. Oocytes were exposed overnight to increasing concentrations (1, 10, 50 & 100 µM) of each compound (**9** in dark blue, **6** in light blue, **10** in red, **7** in pink, **11** in dark green and **8** in light green), in the presence of progesterone. Maturation accomplishment was assessed by the appearance of a white spot at the oocyte animal pole (metaphase II picture) and each mature oocyte was cut (hemi-sections pictures) to confirm the GVBD occurrence. Significance was assessed by ANOVA followed by a Dunnett’s test (***: *p* < 0,001; **: *p* < 0,01; * *p* < 0,05). N refers to the number of females and n to the number of oocytes (*N* = 3 and *n* = 60).

Meiotic resumption in *Xenopus* oocytes was monitored by the appearance of a white spot corresponding to GVBD at the animal-pigmented apex and resulting from the migration of the germinal vesicle to the plasma membrane pushing aside the pigments. Dihydroquinoline derivatives **8, 9** and **10** appeared to exert no effect on GVBD in comparison to the solely progesterone treated control (Figure 3). In contrast, dihydroquinolines **6** and **7** significantly inhibited the occurrence of the white spot in a dose-dependent manner. Compound **7** was more efficient than compound **6** (Figure 4) decreasing GVBD to 68%, 48%, 18%, 2% and 66.6%, respectively and 66.6%, 53.3%, 13.3% for the concentrations of 1, 10, 50 and 100 µM. To further confirm this effect, western blots were performed on samples after 24 h of incubation with the various compounds at the concentration of 100 µM, in presence of progesterone. In metaphase II arrested oocytes, Erk2 and Rsk were phosphorylated and Mos was synthetized to high levels in all treated conditions compared to the progesterone treated oocytes (Figure 3). In oocytes submitted to a treatment with 1,4-dihydroquinolines, Cdk1 was dephosphorylated and cyclin B was present except for the oocytes treated with compounds **6** and **7** were cyclin B was absent. Consequently, Histone H3—a target of the Cdk1/cyclin B active complex— was phosphorylated under the treatment conditions with compounds **8, 9, 10** and **11** but not by compounds **6** and **7**. Two apoptosis markers, the cleaved caspase 3 and the phosphorylated histone H2, were detected in oocytes incubated with compounds **6** and **7**.

### 2.5. Effects of 1,4-Dihydroquinolines on the Meiosis Spindle

**Figure 5:**
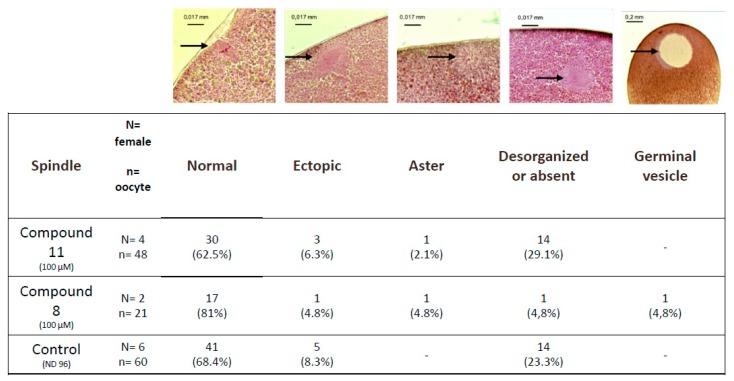
Observed abnormalities in white spot oocytes after ferrocenyl 4-(alkyl-amino)-1,4-dihydroquinolines exposures. Oocytes were exposed to compound **8** and **11** (100 µM) during meiosis resumption. Typical pictures of the observed structures are shown (black arrows) and summarized in the table. N refers to the number of females and n to the number of oocytes.

Untreated metaphase II-arrested oocytes are characterized by a metaphase spindle anchored to the plasma membrane (Figure 5). In oocytes treated with organometallic compound **8** or with control compound **11** the percentages of normal spindles are respectively 81% and 62.5% and are not significantly different compared to the untreated control 68.4%. Ectopic spindles that are not attached to the plasma membrane are present with a low percentage in oocytes treated with compound **8** or with compound **11** (4.8% and 6.3%, respectively) compared to untreated oocytes (8.3%).

The percentage of disorganized or absent spindles in oocytes treated with compound **8** was lower (4.8%) compared to compound **11** and untreated controls (23.3% and 21.1%, respectively). Asters are present in treated oocytes with a very low percentage close to 1%. Oocytes displaying a germinal vesicle are only seen under compound 11 treatment (4.8%).

### 2.6. Maturation Success in 1,4-Dihydroquinolines Pre-Treated Oocytes

**Figure 6:**
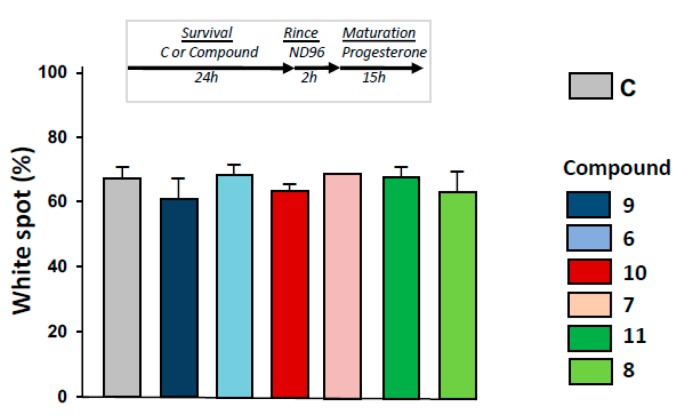
Determination of metaphase II entry in oocytes pre-exposed to ferrocenyl 4-(alkylamino)-1,4-dihydroquinolines. After incubation or not with compounds **9, 6, 10, 7, 11, 8** for 24 h, oocytes were rinsed four times in ND96 for 30 min, before progesterone stimulation. White spot appearance was scored after 15 h. N refers to the number of females and n to the number of oocytes (*N* = 2 and *n* = 60).

As depicted in Figure 6, progesterone-induced metaphasis II entry was not modified significantly by 24 h pre-treatments with 1,4-dihydroquinolines (Figure 6) after oocytes were rinsed for 2 h.

### 2.7. Effects of 1,4-Dihydroquinolines on Oocyte Parthenogenetic Activation

**Figure 7:**
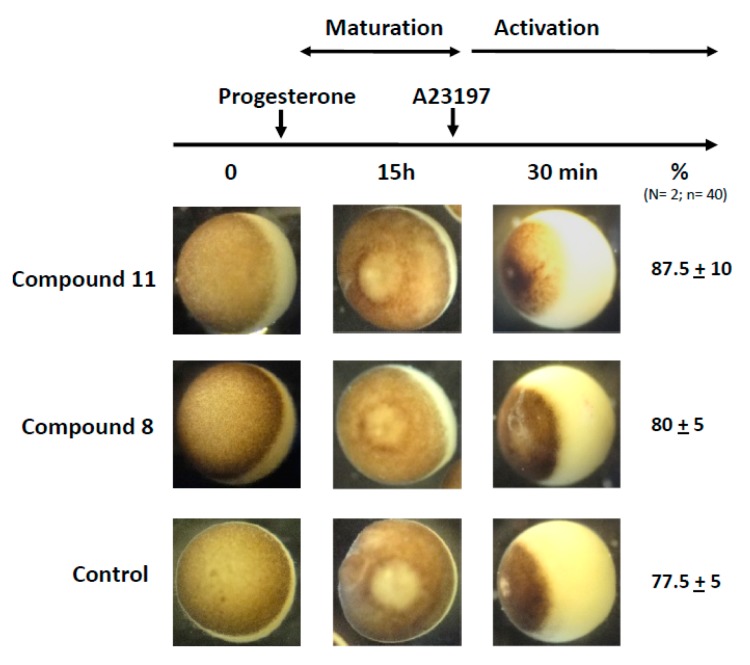
Partenogennetic activation of metaphase II oocytes treated by ferrocenyl 4-(alkylamino)-1,4-dihydroquinolines. Oocytes were incubated or not with compounds **8** or **11** before they were transfered to a 5×-diluted ND96 solution added with A23187 calcium ionophore (50 μM). The numbers of cortical retraction were scored. N refers to the number of females and n to the number of oocytes (*N* = 2 and *n* = 40).

In a last set of experiments, we assessed the capability of the treated oocyte to undergo fertilization. Metaphase II exit can be mimicked by the external addition of A23187 calcium ionophore. As depicted in Figure 7, treatments with progesterone and 1,4-dihydroquinolines **8** or **11** followed by a stimulation with A23187 (50 μM for 30 min) gave rise to a cortical retraction percentage close to that of the progesterone control (Figure 7).

## 3. Discussion

Metal-based strategies are developed to be more effective, less toxic and to overcome intrinsic or acquired resistances. Quinoline has a heterocyclic structure and displays a broad range of biological activities including antimicrobial and antitumor effects. Results presented in this paper show that the presence of a ferrocenyl moiety on dihydroquinolines lowers the IC_50_ to 1.88 and 6.77 μM, in two cancer cell lines. This data suggests a 20-fold increase in effectiveness compared to values previously reported for ferrocene complexes and is comparable to the range of active concentrations of other anticancer agents on cell lines (nM to μM) [23,28,38]. The ferrocene group possesses unique electronic and structural characteristics and ferrocene derivatives exhibit enhanced biological properties [23,24]. The antiproliferative effects of ferrocenyl are associated with redox properties and ROS (reactive oxygen species) production [23,24]. Ferrocenyl complexes react with hydrogen peroxide and generate hydroxyl radicals, toxic forms of ROS, capable of damaging DNA and other cellular constituents.

The increasing use of pharmaceuticals results in increased discharges and aquatic contamination [35], for example in Brazil doses up to 123.5 μg/L were measured [39], but higher concentrations all over the world were also reported (see [40] for a review). *Xenopus* oocytes (M-Phase entry and meiotic maturation from prophase I to metaphase II) offer a unique physiological aquatic model to assess the effects of novel synthesized products on cell cycle. In our experiments, no effects were observed on prophase I oocytes treated with 1,4-dihydroquinolines **6**, **8**, **9**, **10** and **11**. Furthermore, their metaphase II entry could be triggered by progesterone. On the contrary, compound **7** appeared to dramatically impair oocyte survival. No cell volume modification was observed in accordance with the fact that *Xenopus* oocyte apoptosis is not associated with cell shrinkage [41,42,43]. In addition, compounds **6** and **7** affect the resumption of oocyte meiosis triggered by progesterone. It was previously reported that apoptosis occurred in progesterone-treated oocyte 48 h after the completion of maturation [39,40]. Two apoptosis markers, the cleaved caspase 3 [44,45] and the Ser14 phosphorylated histone H2B [44,45,46,47] were detected in prophase I oocytes exposed to compound **7** and in oocytes treated by progesterone and compounds **6** or **7** showing an apoptosis death. Cleaved caspase 3 is an early apoptotic marker, but given that the overall duration of apoptosis is 48 h, no altered phenotypes were observed in oocytes after 24 h of treatment.

**Figure 8:**
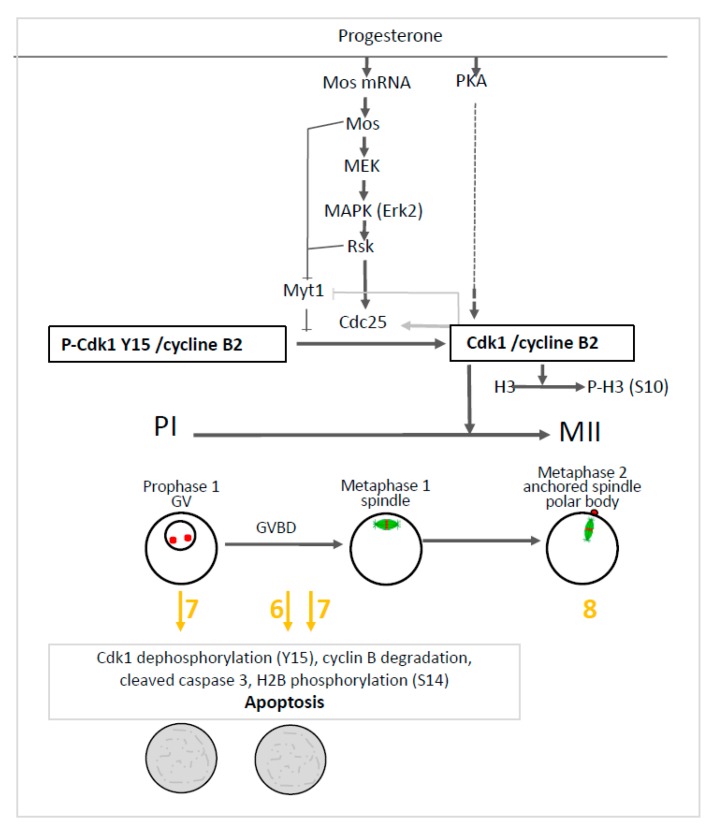
Effect of ferrocenyl 4-(imino)-1,4-dihydroquinolines on oocytes arrested in prophase I (PI) and oocytes induced to progress in metaphase II (MII). Progesterone activates PKA, Mos synthesis and the MAPKK/MAPK/Rsk cascade. The two pathways further activate phosphatase Cdc25, inhibit kinase Myt1 and converge to activate the Cdk1/cyclin B complex. Rsk, Mos and the activated Cdk1/cyclin B complex inhibit Myt1. The activated Cdk1/cyclin B complex also activates Cdc25 and phosphorylates histone H3. The germinal vesicle (GV) of prophase I oocytes is disrupted (GVBD) upon progesterone addition and the oocytes progress into metaphase I and metaphase II. The nuclear spindle (microtubules-green and chromosomes-red) is formed in metaphase I, is attached to the plasma membrane in metaphase II and accompanied by the emission of a polar body. Ferrocenyl 4-(imino)-1,4-dihydroquinolines **6, 7** (in yellow/orange) induce Cdk1 dephosphorylation, cyclin B degradation, caspase 3 cleavage and histone H2B phosphorylation leading to oocytes apoptosis after meiosis resumption has been triggered by progesterone addition (grey rectangle). Compound **7** produces the same effect on prophase I arrested oocytes. Compound **8** (in yellow/orange) did not affect oocyte progression into metaphase II.

To get insight into the effect induced by compounds **6** and **7**, we further investigated the main molecular effectors involved in oocytes meiosis. In prophase I and metaphase II oocytes treated by the organometallic compounds, the Mos/MAPK/Rsk cascade was not altered. Erk2 and Rsk were phosphorylated and Mos was synthesized. Only compound **7** triggered the dephosphorylation of Cdk1 and the total degradation of cyclin B in prophase I oocytes. The same effect was observed in progesterone treated oocytes with compounds **6** and **7**. Meiosis progression requires two waves of MPF activity at meiosis I and II. Cdk1 dephosphorylation, Cdc25 activation, Myt1, and a MAPK auto-amplification loop are also involved in oocyte maturation (see Figure 8). In fully-grown stage VI oocytes, a pool of inactive preformed MPF is present [48]. Under progesterone activation, the pre-MPF activation requires new protein synthesis. Compounds **6** and **7** allowed some protein synthesis as Mos was synthesized. The preformed MPF stock is degraded after meiosis I and consequently requires new cyclin B synthesis to reach meiosis II [48]. However, when cyclin B synthesis is experimentally suppressed, oocytes degenerate [48] as observed for oocytes treated with compounds **6** and **7**. Cyclin B degradation and MPF inactivation were reported to be separate events and a non-proteolytic activity of the proteasome protects Cdk1 (complexed to cyclin B) from degradation [49,50]. In oocytes undergoing apoptosis, it was demonstrated that Cdk1 dephosphorylation and the total cyclin B degradation are associated with active caspase 3 formation and H2B phosphorylation [44,45,46,47]. The same effects were observed in oocytes treated with progesterone and compounds **6** or **7**. These compounds nevertheless act at different levels in the oogenesis process. Compound **6** is associated with Cdk1 dephosphorylation and cyclin B degradation only when meiosis resumption is triggered whereas compound **7** also acts in prophase I-arrested oocytes (see Figure 8).

Only compound **8** displays no significant effect on oocyte survival and maturation nor did it impact oocytes membrane potential and calcium-induced currents (Supplementary Figures S1 and S2). As quinolines have been reported to affect spindle structure [51] and quinazolinones to be selective inhibitors of the kinesin spindle protein [52], we therefore investigated the effect of compound **8** on the metaphase II spindle. After the first polar body has formed, the second meiotic spindle is placed underneath the oocyte surface and rotates into its final axial position [53]. Compound **8** did not alter the spindle organization and normal spindles were detected correctly anchored to the membrane. The percentage of abnormal spindles observed in controls is not surprising and is comparable to previous reports [13]. Moreover, parthenogenetic activation enabled a typical cortical retraction indicating that calcium activation mimics the fertilization event in compound **8**-treated oocytes. Experiments performed on early development showed compound 8 could not alter the embryo early cleavage and gastrulation (Supplementary Figure S3).

Finally, ferrocenyl compound **8** appears as a good antiproliferative drug with no impact on amphibian oogenesis. However further works certainly needs to be undertaken on early embryological stages to ascertain its apparent harmless effect.

## 4. Materials and Methods

### 4.1. General Information

All commercial reagents and solvents were used without further purification. Melting points were determined with an Electrothermal (BI 9300) capillary melting point apparatus (Thermo Fisher Scientific, Waltham, MA) and are uncorrected. The ^1^H- and ^13^C-NMR spectra were recorded on an AC300 spectrometer (Bruker, Billerica, MA) at 300 and 75.5 MHz respectively using tetramethylsilane (TMS) as internal standard and CDCl_3_ as solvent. Mass spectra were recorded with a LCMS-MS triple-quadrupole system (1200ws, Varian, Palo Alto, CA). Thin layer chromatography (TLC) was carried out on aluminium-baked silica gel 60 (Macherey-Nagel, Hoerdt, France). Column chromatography was performed on silica gel (230–400 mesh). Elemental analyses were performed with a vario MICRO analyser (Elementar, Lyon, France).

### 4.2. Synthesis of 7-Chloro-1-Ferrocenylmethyl-4-(Benzylimino)-1,4-Dihydroquinoline (***6***)

To a solution of 4,7-dichloroquinoline (**1**, 116 mg, 0.585 mmol) and PTSA (117 mg, 0.615 mmol) in CH_3_CN (5 mL), ferrocenylmethanol (**2,** 100 mg, 0.463 mmol) was added and stirred for 40 min at rt, then benzylamine (**3**, 150 µL, 1.37 mmol) was added to the reaction mixture. After stirring for 40 min at rt, the mixture was hydrolyzed with KOH aqueous solution (5 mL) and extracted with CH_2_Cl_2_ (3 × 20 mL). The organic layer was washed with water, dried over anhydrous sodium sulfate and evaporated under reduced pressure. The crude product was purified on a silica gel column using EtOAc/NEt_3_: 90/10 as eluent to afford **6** as yellow powder (0.164 mg, 76%). ^1^H-NMR (CDCl_3_, 300 MHz): δ 8.57 (d, *J* = 8.7 Hz, 1H), 7.3 (m, 7H), 6.98 (d, *J* = 8.1 Hz, 1H), 6.00 (d, *J* = 8.1 Hz, 1H), 4.76 (s, 2H), 4.59 (s, 2H), 4.23 (m, 9H). ^13^C-NMR (CDCl_3_, 75 MHz): δ 154.8, 141.8, 139.5, 137.9, 136.1, 128.2, 127.6, 127.4, 126.2, 124.1, 123.4, 114.3, 99.4, 81.7, 68.9, 68.8, 68.8, 53.4, 51.1. LC/MS: MH^+^= 466.89.

### 4.3. Synthesis of 7-Chloro-1-Ferrocenylmethyl-4-(Pentylimino)-1,4-Dihydroquinoline (***7***)

To a solution of 4,7-dichloroquinoline (**1**, 116 mg, 0.585 mmol) and PTSA (117 mg, 0.615 mmol) in CH_3_CN (5 mL), ferrocenylmethanol (**2**, 100 mg, 0.463 mmol) was added and stirred for 40 min at rt, then amyl amine (**4**, 162 µL, 1.39 mmol) was added to the reaction mixture. After stirring for 40 min at rt, the mixture was hydrolyzed with KOH aqueous solution (5 mL) and extracted with CH_2_Cl_2_ (3 × 20 mL). The organic layer was washed with water, dried over anhydrous sodium sulfate and evaporated under reduced pressure. The crude product was purified on a silica gel column using EtOAc/NEt_3_: 90/10 as eluent to afford **7** as yellow powder (163 mg, 79%). ^1^H-NMR (CDCl_3_, 300 MHz): δ 8.47 (d, *J* = 8.6 Hz, 1H), 7.27 (d, *J* = 1.7 Hz, 1H), 7.17 (dd, *J* = 8.6 and 1.7 Hz), 7.02 (d, *J* = 8.0 Hz, 1H), 5.97 (d, *J* = 8.1 Hz, 1H), 4.77 (s, 2H), 4.22, 4.23 (m, 9H), 3.30 (t, *J* = 7.3 Hz, 2H), 1.72 (q, *J* = 7.1 Hz, 2H), 1.43 (m, 4H), 0.93 (t, *J* = 7.1 Hz, 3H). ^13^C-NMR (CDCl_3_, 75 MHz): δ 153.8, 139.5, 137.9, 136.2, 127.2, 123.5, 114.3, 99.1, 81.7, 68.9, 68.8, 68.7, 51.2, 49.6, 30.6, 30.0, 22.7, 14.2.

### 4.4. Synthesis of 7-Chloro-1-Ferrocenylmethyl-4-(Phenylylimino)-1,4-Dihydroquinoline (***8***)

To a solution of 4,7-dichloroquinoline (**1**, 116 mg, 0.585 mmol) and PTSA (117 mg, 0.615 mmol) in CH_3_CN (5 mL), ferrocenylmethanol (**2**, 100 mg, 0.463 mmol) was added and stirred for 40 min at rt, then aniline (**3**, 125 µL, 1.37 mmol) was added to the reaction mixture. After stirring for 40 min at rt, the mixture was hydrolyzed with KOH aqueous solution (5 mL) and extracted with CH_2_Cl_2_ (3 × 20 mL). The organic layer was washed with water, dried over anhydrous sodium sulfate and evaporated under reduced pressure. The crude product was purified on a silica gel column using EtOAc/NEt_3_: 90/10 as eluent to afford **8** as yellow powder (172 mg, 82%). ^1^H-NMR (CDCl_3_, 300 MHz): δ 8.56 (d, *J* = 8.7 Hz, 1H), 7.38 – 7.32 (m, 3H), 7.25 (dd, *J* = 8.7, 1.8 Hz, 1 H), 7.05 (t, *J* = 7.4 Hz, 1H), 6.96-6.92 (m, 3H), 5.93 (d, *J* = 8.1 Hz, 1H), 4.79 (s, 2H), 4.24 (sb, 9H). ^13^C-NMR (CDCl_3_, 75 MHz): δ 154.1, 152.7, 140.1, 138.3, 136.8, 129.2, 127.9, 123.8, 123.7, 122.2, 121.3, 114.4, 101.2, 81.6, 68.9, 68.8, 68.7, 51.3.

### 4.5. Synthesis of 1-Benzyl-7-Chloro-4(Benzylimino)-1,4-Dihydroquinoline (***9***)

Compound **9** was synthesized according to the method of Surrey. A mixture of 4,7-dichloroquinoline (**1**, 198 mg, 1 mmol), benzyl chloride (253 mg, 2 mmol) and sodium iodide (900 mg, 6 mmol) in acetone (5 mL) was refluxed for 12 h with stirring. The reaction mixture was allowed to cool and the resulting precipitate was collected and washed with water and acetone. The resulting solid was added to a solution of benzylamine (321 mg, 3 mmol) in ethanol (5 mL). After stirring under reflux for 1 h, the mixture was hydrolysed with KOH aqueous solution (5 mL) and extracted with CH_2_Cl_2_ (3 × 20 mL). The organic layer was washed with water, dried over anhydrous sodium sulfate and evaporated under reduced pressure. The product was obtained as a green powder (70 mg, 20 % for the two steps). M.p. 212°C. ^1^H-NMR (CDCl_3_, 300 MHz) δ 8.50 (d, *J* = 8.7 Hz, 1H), 7.40 (d, *J* = 7.5 Hz, 1H), 7.28-7.05 (m, 9H), 6.94 (d, *J* = 3.9 Hz, 1H), 6.01 (d, *J* = 8.0 Hz, 1H), 4.96 (s, 2H), 4.54 (s, 2H).

### 4.6. Synthesis of 1-Benzyl-7-Chloro-4(Pentylimino)-1,4-Dihydroquinoline (***10***)

Compound **10** was synthesized according to the method of Surrey. A mixture of 4,7-dichloroquinoline (**1**, 100 mg, 0.5 mmol), benzyl chloride (115 µM, 1.0 mmol) and sodium iodide (450 mg, 3.0 mmol) in acetone (5 mL) was refluxed for 12 h with stirring. The reaction mixture was allowed to cool and the resulting precipitate was collected and washed with water and acetone. The resulting solid was added to a solution of pentylamine (50 µL, 0.431 mmol) in ethanol (5 mL). After stirring under reflux for 1 h, the mixture was hydrolyzed with KOH aqueous solution (5 mL) and extracted with CH_2_Cl_2_ (3 × 20 mL). The organic layer was washed with water, dried over anhydrous sodium sulfate and evaporated under reduced pressure. The product was obtained as a green powder (47 mg, 27 % for the two steps). ^1^H-NMR (CDCl_3_, 300 MHz): δ 9.18 (d, *J* = 9.0 Hz, 1H), 8.51 (d, *J* = 7.5 Hz, 1H) 7.57 (s,1H), 7.39 (d, *J* = 9.3 Hz, 1H), 7.29 (m, 3H), 7.13 (m, 2H), 6.56 (d, *J* = 7.5 Hz, 1H), 5.66 (s, 2H), 3.53 (q, *J* = 7.2 Hz, 2H), 1.74 (m, 2H), 1.30 (m, 4H), 0.81 (t, *J* = 7.05 Hz, 3H).

### 4.7. Synthesis of 1-Benzyl-7-Chloro-4(Phenylimino)-1,4-Dihydroquinoline (***11***)

A solution of 4,7-dichloroquinoline (**1**, 396 mg, 2 mmol) and aniline (218 mg, 2.34 mmol) was placed in an oven-dried test tube equipped with a magnetic stir bar and a Teflon screw-cap without solvent. The reaction mixture was then irradiated in a closed vessel monomode microwave at 100 °C for 10 min. After cooling to ambient temperature, an aqueous solution NaOH 1 M was added and the reaction mixture was extracted with ethyl acetate (2 times). The combined organic layers were washed with water then dried over MgSO_4_ and evaporated under reduced pressure. 360 mg of white powder were obtained and this compound was used without any further purification. To the resulting solid benzyl chloride (180 mg, 1.42 mmol) was added in an oven-dried test tube equipped with a magnetic stir bar and a Teflon screw-cap using acetonitrile (1 mL) as solvent. The reaction mixture was then irradiated in a closed vessel monomode microwave at 80 °C for 40 min. After cooling to ambient temperature, an aqueous solution NaOH 1M was added and the reaction mixture was extracted with ethyl acetate (2 times). The combined organic layers were washed with water then dried over MgSO_4_ and evaporated under reduced pressure to furnish 250 mg of yellow powder. The purification by chromatography over silica gel (ethyl acetate) afforded pure compound **11** as yellow powder (122 mg, 28%). ^1^H-NMR (CDCl_3_, 300 MHz): δ 8.53 (d, *J* = 9.0 Hz, 1H), 7.36 (m, 5H); 7.19 (m, 3H); 7 (m,5H); 5.98 (d, *J* = 9.0 Hz, 1H); 5.05 (s, 2H). ^13^C-NMR (CDCl_3_, 75 MHz): 154.1, 152.6, 140.2, 139.8, 137, 135.3, 129.3, 129.2, 128.2, 127.8, 126.1, 123.9, 122.3, 121.3, 114.9, 101.5, 55.6.

### 4.8. Reagents and Substances for Biological Assays

The purity of compounds was of molecular biology grade. They were purchased from Sigma-Aldrich Chimie (Saint-Quentin Fallavier, France) unless otherwise specified. Solutions were prepared daily by appropriate dilutions in ND96 medium (in mM: NaCl 96, KCl_2_, MgCl_2_ 1, CaCl_2_ 1.8, HEPES 5, adjusted to pH 7.5 with NaOH).

### 4.9. Frog and Oocyte Handling

*Xenopus laevis* females were obtained from the CRB-University of Rennes I, France, and housed at the University of Lille-PHExMAR. Females were anesthetized by an immersion in a solution of tricaine methane sulfonate (MS222, Sandoz, Holzkirchen, Germany) at 1 g·L^−1^ for 45 min and ovaries were surgically removed and placed in ND96 medium. Stage VI oocytes were harvested by using a 1 h collagenase A treatment (1 mg/mL, Boehringer Mannheim, Grenoble, France) for 45 min and was achieved by a manual dissociation under a binocular microscope. The oocytes were kept in ND96 medium at 19°C. Laboratory animal experimentations were performed according to the European Community Council guidelines (86/609/EEC). The protocols were approved by the institutional local “Comité d’Ethique et d’Expérimentation Animale, Région Haut de France, F59-00913”.

### 4.10. 1,4-Dihydroquinoline Exposure, Oocyte Death, GVBD Detection and Oocyte Parthenogenetic Activation

For oocyte survival and meiosis resumption experiments, oocytes were exposed for 24 or 48 h at 19 °C to 1,4-dihydroquinoline solutions (compounds **6**-**11**) at concentrations of 1 µM, 10 µM, 50 µM or 100 µM. Phenotypically, the unhealthy and dying oocytes were detected by an unorganized rearrangement of the homogenous animal pigmentation and a general bleaching. Meiotic resumption was triggered by the addition of progesterone (4 µg·mL^−1^) alone or concomitantly or after 1,4-dihydroquinolines. The appearance of a white spot at the apex of the pigmented hemisphere of the oocyte indicated the ongoing meiosis process including migration and dissolution of the germinal vesicle (germinal vesicle breakdown, GVBD) and followed by the formation and the attachment of the meiotic spindle to the plasma membrane, characteristic of metaphase II arrested oocyte. In some cases, oocytes were heat fixed at 100 °C for 5 min and hemi-sections along the animal to the vegetative pole were performed to ascertain the presence of the germinal vesicle. As control, staurosporine (20 mM) was used to induce prophase I oocytes apoptosis [46]. For parthenogenetic activation, in vitro-matured oocytes previously treated by progesterone (4 µg·mL^−1^), with or without compounds **8** or **11**, for 24 h, were transferred to a solution of five times water diluted ND96 containing 50 μM of A23187 calcium ionophore (Calbiochem^®^, Millipore Corporation, Burlington, MA). The typical cortical reaction visualized by a pigment retraction towards the animal region was monitored after 20 to 30 min.

### 4.11. Electrophoresis and Western Blot Analysis

Oocytes were lysed at 4 °C in the following homogenization buffer (10 µL/oocyte): 500 mM NaCl, 0.05% SDS, 0.5% Triton X100, 5 mM MgCl_2_, 1 mg/mL bovine serum albumin, 10 μg/mL leupeptin, 10 μg/mL aprotinin, 10 μg/mL soybean trypsin inhibitor, 10 μg/mL benzamidine, 1 mM PMSF, 1 mM sodium vanadate, 50 mM HEPES at pH 7.4. After a centrifugation for 15 min at 10,000× *g* (4 °C), to eliminate the yolk and the membranous pellet, the supernatant fraction was mixed with Laëmmli sample buffer containing 4% β-mercaptoethanol (BioRad, Hercules, CA) and heated at 100 °C for 3 min. Proteins were electrophoresed on SDS–PAGE gels (BioRad gradient from 4 to 20%) and transferred to a Hybond ECL membrane (Amersham Life Sciences, Little Chalfont, UK) in Tris/NaCl/Tween/BSA pH8 (15mM Tris HCl, 150 mM NaCl, 0.1% tween, 10% bovine serum albumin, Sigma-Aldrich, Saint-Louis, MO). The transfer efficiency was checked using a Ponceau red reversible coloration (0.2% in 3% trichloracetic acid, Sigma). For western blotting, the membranes were cut according to the molecular weight of the protein to be detected. Membranes were immunoblotted and when necessary stripped with a ready to use stripping buffer (Clinisciences, Nanterre, France). Membranes were saturated with a solution of TBS-Tween (50 mM Tris, 150 mM NaCl, 0.1% (*v*/*v*) Tween20, pH7.4) added with 5% (*w*/*v*) non-fat milk for 45 min before they were incubated at 4 °C overnight with rabbit polyclonal antibodies against cleaved caspase 3 (Cell Signaling Technology, Danvers, MA, 1/1000), Cdk1 (17, Santa Cruz Biotechnology, Dallas, TX, 1/1500), anti-phosphorylated histone H2B (Upstate Biotechnology Inc., Boston, MA, 1/1000), phosphorylated histone H3 (S10, Cell Signaling, 1/1500), Rsk1 (sc231, Santa Cruz Biotechnology Inc.,1/1000), phosphorylated Rsk (S380, Santa Cruz Biotechnology, 1/1000), or mouse monoclonal antibodies against Tyr15 phosphorylated Cdk1 (Y15, Cell Signaling, Danvers, MA, 1/1500), Cyclin B2 (sc53239, Santa Cruz Biotechnology, 1/1500), Erk2 (D-2 sc1647, Santa Cruz Biotechnology, 1/1500), Tyr 202/204 phosphorylated Erk2 (Y202:Y204, Santa Cruz Biotechnology, 1/1000), Mos (c237, Santa Cruz Biotechnology, 1/500) or goat polyclonal antibody against actin (Santa Cruz Biotechnology, 1/1500). Primary antibodies were washed three times 10 min in TBS-Tween and incubated 1 h with either an anti-rabbit or an anti-mouse horseradish peroxidase-labeled secondary antibody (Invitrogen, Frederick, MD, USA, 1:30,000). The detection was performed with an enhanced chemiluminescence system (Advanced ECL Detection system, Amersham) on hyperfilms (Amersham).

### 4.12. Histological Detection of Metaphase II Spindle

Oocytes exhibiting a white spot were collected after an overnight exposure or not at 19 °C, fixed overnight in the dark at room temperature with Smith reagent composed by potassium bichromate 17 mM added with formol and acetic acid 80/20%. Oocytes were ethanol dried, paraffin-embedded, and sliced with a microtome (7 μm). Colorations with nuclear red (0.1 g of nuclear red QSP in 100 mL of aluminium sulphate 5%) was conducted to reveal the chromosomes, and picro-indigo-carmine (0.25 g of picro-indigo-carmine QSP in 100 mL of saturated picric acid) to detect the cytoplasmic structures [54]. Slices were mounted with Entellan^®^ mounting medium (Merck Millipore, Fontenay sous Bois, France) before they were analysed under light microscope (×400, ICC50, Leica, Wetzlar, Germany).

### 4.13. Cell Culture and Antiproliferative Activity Analysis

Cell lines MDA-MB-231 from triple-negative breast cancer and HeLa from cervical cancer were obtained from the American Type Culture Collection (ATCC; Rockville, MD, USA). They were cultured at 37 °C with 5% CO_2_ in DMEM middle (Lonza, Basel, Switzerland), supplemented with 10% fetal bovine serum (Dutscher, Brumath, France), 1% Zell Shield (Dutscher) and 1% non-essentials amino-acids (Lonza). Cancer cells were plated in 96-well plates (2 × 10^3^ cells/wells) in triplicate, incubated at 37 °C with 5% CO_2_ for 24 h, and treated with 1,4-Dihydroquinolines for 72 h at different concentrations. Cell viability was determined using CellTiter 96^®^ AQueous One Solution Cell Proliferation Assay (MTS, Promega, Madison, WI, USA). 20 µL reagent was added in each well and cells were incubated for 2 h at 37 °C/5% CO_2_. Absorbance was measured at 490 nm (SPECTROstar Nano, BMG LABTECH, Ortenberg, Germany). Calculations of IC50 were performed using GraphPad Prism V7.0 software (GraphPad Software, La Jolla, CA, USA).

### 4.14. Statistical Analysis

Experiments were performed on 3 different females in triplicate and on 10 to 20 oocytes per female. The results are shown as mean +/− standard error of the mean (SEM). For statistical analysis, a two-way ANOVA followed by Dunnett’s test was performed. Statistical significance was accepted for * *p* < 0.05, ** *p* < 0.01 and *** *p* < 0.001.

## 5. Conclusions

The use of *Xenopus* oocytes enables the molecular action of pharmacological compounds to be deciphered and allow scientists to address the complex relationships between human health and environmental factors. Ferrocene compound **8** shows an antiproliferative effect at low doses on human cancer cells, with no effect on xenopus survival and meiotic maturation.

## Figures and Tables

**Table 1:** Determination of IC_50_ values on two cancer cell lines. Cancer cells (HeLa & MDA-MB 231) were exposed to 4-(imino)-1,4-dihydroquinoline derivatives and cell viability was determined after 72 h using Cell Proliferation Assay (MTS). IC_50_ values were determined on three independent experiments.

Compounds	IC_50_ Hela (μM)	IC_50_ MDA-MB-231 (Μm)
**9**	18.28 ± 7.95	26.03 ± 2.94
**6**	6.70 ± 1.11	8.06 ± 1.00
**10**	14.25 ± 7.28	11.04 ± 0.29
**7**	4.91 ± 0.04	3.39 ± 1.52
**11**	42.09 ± 2.87	13.61 ± 0.47
**8**	6.21 ± 1.73	7.39 ± 0.19

## Supplementary Materials

Supplementary materials can be found at https://www.mdpi.com/1422-0067/21/9/3049/s1.

## Abbreviations

FETAX	Frog Embryo Teratogenesis Assay Xenopus
GVBD	Germinal Vesicle BreakDown
IC50	half maximal inhibitory concentration
MPF	M-phase Promoting Factor
MS222	Tricaïne Methane Sulfonate
MTT	3-(4,5-dimethylthiazol-2-yl)-2,5-diphenyltetrazolium bromide
