# Supplementary material for: Effects of Ferrocenyl 4-(Imino)-1,4-Dihydro-quinolines on Xenopus laevis Prophase I - Arrested Oocytes: Survival and Hormonal-Induced M-Phase Entry
Source: Int J Mol Sci. 2020 Apr 26;21(9):3049. doi: 10.3390/ijms21093049 (PMC7246863; doi:10.3390/ijms21093049)
Supplement: Supplementary file 1 [file ijms-21-03049-s001.pdf]

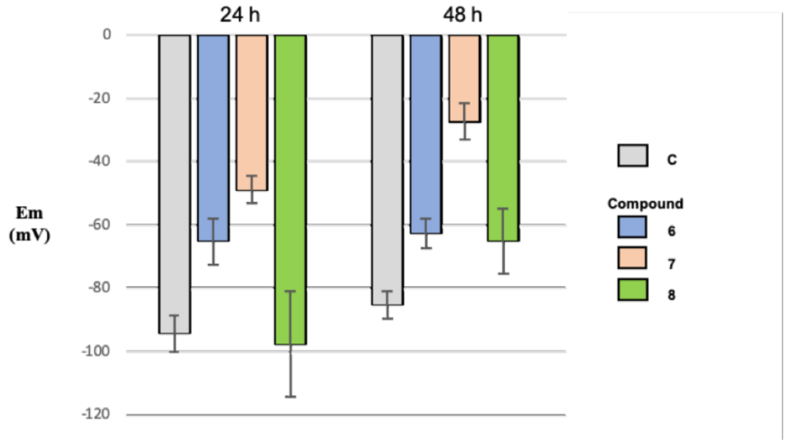

Supplementary Figure S1. membrane potential (Em) recorded from oocytes treated or not with compounds 6, 7 and 8 for 24 h, 48 h and 72 h. Records were performed using the double electrode technique and Clampex 6.0 software (Invitrogen, Saint-Aubin, France). Results are expressed as mean ± SEM.

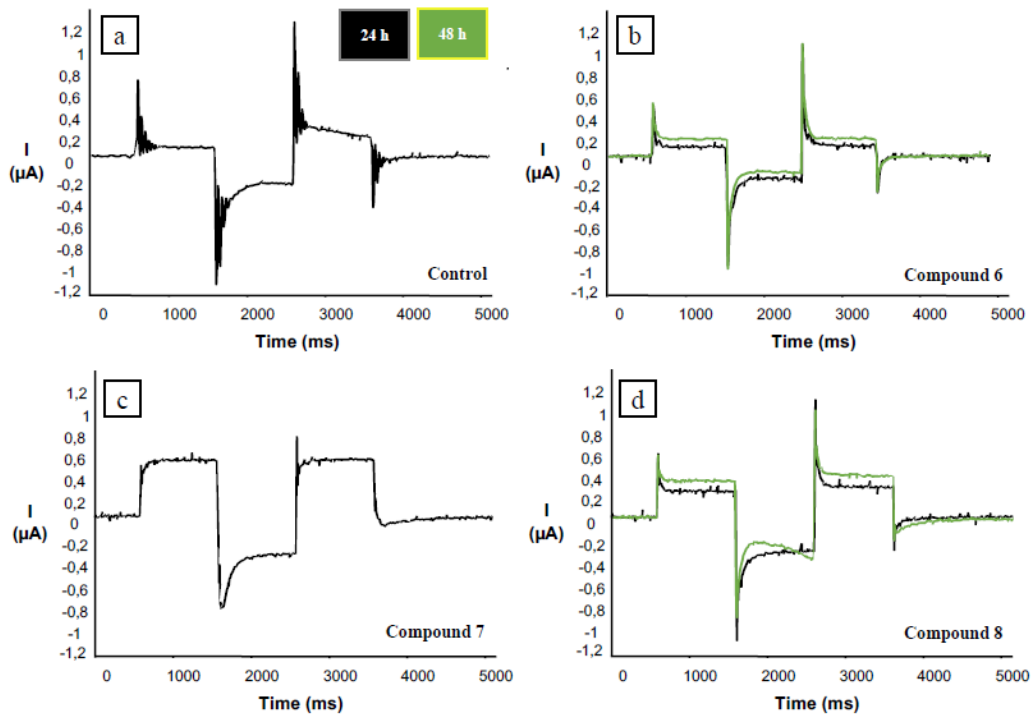

Supplementary Figure S2. Calcium-activated chloride currents (ICl) in oocytes exposed or not (a) to compounds **6** (b), **7** (c), and **8** (d). ICl currents were evoked using a triple step protocol after 24 h (black), 48 h (green) and 72 h (red), Control oocyte recording is shown for 24 h (the same currents were observed at 48 h and 72 h). Oocytes treated with compound 7 dyed after 24 h. The "triple step" protocol (developed by Y. Yao, R. Y. Tsien, *J. Gen. Physiol.* **1997**, *109*, 703–715) consisted in a first depolarization from -40 mV to +40 mV, followed by a hyperpolarization to -140 mV and a third depolarizing step at +40 mV. ICl1-S is measured at the end of the first depolarisation and represent the ionic current activated by internal calcium release. ICl2 obtained at the end of the second hyperpolarizing step, mirrored extracellular calcium entry. ICl1-T the third measured current is the "a tail current" activated by both intracellular calcium release and extracellular calcium entry. The recordings are representative of at least 9 different oocytes from at least 3 different females.

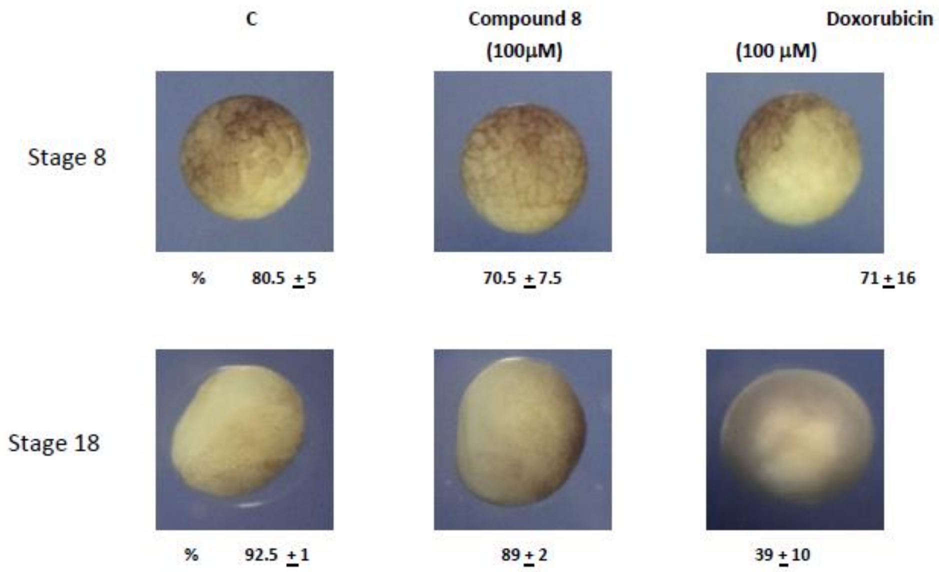

Supplementary Figure S3. *Xenopus* embryos were obtained by artificial fertilization and maintained in water with or without compound 8 (100 mM) or doxorubicin (100 mM). Embryos were staged according to Nieuwkoop and Faber table.
